# Clustering PPI data by combining FA and SHC method

**DOI:** 10.1186/1471-2164-16-S3-S3

**Published:** 2015-01-29

**Authors:** Xiujuan Lei, Chao Ying, Fang-Xiang Wu, Jin Xu

**Affiliations:** 1 School of Computer Science, Shaanxi Normal University, Xi'an, Shaanxi 710062, China; 2 School of Electronics Engineering and Computer Science, Peking University, Beijing,100871, China; 3 Division of Biomedical Engineering, University of Saskatchewan, Saskatoon, SK S7N 5A9, Canada

**Keywords:** Protein-Protein Interaction (PPI) data, firefly algorithm (FA), synchronization-based hierarchical clustering (SHC), spectral clustering (SC)

## Abstract

Clustering is one of main methods to identify functional modules from protein-protein interaction (PPI) data. Nevertheless traditional clustering methods may not be effective for clustering PPI data. In this paper, we proposed a novel method for clustering PPI data by combining firefly algorithm (FA) and synchronization-based hierarchical clustering (SHC) algorithm. Firstly, the PPI data are preprocessed via spectral clustering (SC) which transforms the high-dimensional similarity matrix into a low dimension matrix. Then the SHC algorithm is used to perform clustering. In SHC algorithm, hierarchical clustering is achieved by enlarging the neighborhood radius of synchronized objects continuously, while the hierarchical search is very difficult to find the optimal neighborhood radius of synchronization and the efficiency is not high. So we adopt the firefly algorithm to determine the optimal threshold of the neighborhood radius of synchronization automatically. The proposed algorithm is tested on the MIPS PPI dataset. The results show that our proposed algorithm is better than the traditional algorithms in *precision*, *recall *and *f-measure *value.

## Introduction

Protein-protein interaction(PPI) data [1] have been very important sources in the researches of life science, which can explore biological functions so as to deeply understand the essence of life activities and mechanism of diseases. Clustering analysis of PPI data is an effective way to predict the function modules and protein complex and, study mechanisms, diagnosis and treatment of diseases.

PPI data are often represented as PPI network. Traditional clustering methods do not perform well for PPI data due to the properties of their represented networks such as small world and scale free characters [1,2]. Many new algorithms were proposed for clustering PPI networks [3,4]. In 2002 years, Girvan and Newman[5] proposed a clustering algorithm based on hierarchical divisions, which deletes the edge with the biggest betweenness [6,7] constantly to separate modules. The Newman fast algorithm [8] is a kind of clustering algorithm based on hierarchy condensations, in which the algorithm continually merges two modules that have the highest similarity. Restricted Neighborhood Search Clustering (RNSC) algorithm [9] is another kind of clustering algorithm based on graph partitioning, which starts with a random partition of a network and iteratively moves the nodes on the border of a cluster into the adjacent cluster to search for a better clustering result with the minimum cost. Clique Percolation Method (CPM) was put forward by Palla [10], in which the *k*-cliques was identified by using clique percolation firstly, and then the adjacent *k*-cliques were combined to get the functional modules. Bader *et al*. proposed molecular complex detection (MCODE) [11], in which every node was weighted by the node's local neighbor density firstly, then the nodes with high weights were picked as the seed nodes of initial clusters and further these clusters were augmented to form the preliminary clusters. Markov clustering (MCL) [12] is a graph clustering based on flow simulation, which has been applied to detect functional modules through simulating random walks within a graph. Spectral clustering-based (SC) method [13] converts the problem to a quadratic optimization with constraints by utilizing the methodology of matrix analysis, which is generally applied to the fields of image segmentation and complex network clustering. Some methods advise that we should consider the gene expression data and detect protein complexes basing on uncertain graph model [14,15],There are many new algorithms also, such as Ovrlp, PE-WCC, UVCluster, AP, GFA, ADMSC, SCI-BN, CORE, FAG-EC, HC-PIN, IPCA, CP-DR, LF-PIN, ABC algorithm [16-29] and so on.

Synchronization is a natural phenomenon ranging from the metabolism in the cell to social behavior in groups of individuals regulating a large variety of complex processes. The sync [30] algorithm inherited from synchronization, which is a novel approach to cluster objects inspired by the powerful concept of synchronization. The basic idea is to regard each object as a phase oscillator and simulate their interaction behaviors over time. The similar phase oscillators synchronize together and form distinct clusters naturally along with time increasing. Without depending on any distribution assumptions, the sync algorithm can detect clusters of arbitrary number, shape and size. In addition, because the outliers do not synchronize with cluster objects, the concept of synchronization allows handling the natural outliers. However, the running time of the algorithm is too long to process the large-scale data. The running time of the algorithm consists of two parts primarily: the dynamic interaction time of synchronizing objects and the process of determining the optimal synchronous neighborhood radius. For reducing the dynamic interaction time of synchronization of data, the concept of ε-neighborhood closures was proposed in the synchronization-based hierarchical clustering (SHC) [31,32] algorithm, the objects in a neighborhood closures will reach synchronization completely and eventually form a cluster. So it can detect clusters by putting the objects in the same neighborhood closures to a cluster even if the objects do not synchronize completely. However, the SHC algorithm determines the optimal value of synchronous neighborhood radius by means of hierarchical search that the sync algorithm does. The hierarchical search for the optimal value of synchronous neighborhood radius not only has low efficiency but also has other two shortcomings. The hierarchical search is very difficult to find the optimal value of synchronous neighborhood radius, and the hierarchical incremental Δε needs to be adjusted according to the different object distributions.

Swarm intelligence optimization algorithm is a kind of bionic algorithms developed in recent years, which is characterized by simply handling, collateral implementation and strong robustness. The searching process for the optimal value of swarm intelligence optimization does not require the solution set differentiable or even continuous. So the swarm intelligence optimization algorithm is applied extensively to pattern recognition, automatic control, robot path planning and other fields. The firefly algorithm (FA) [33-35] is an intelligent optimization algorithm developed by simulating the glowing characteristics of fireflies based on group searching. The bionic principle of the FA algorithm is looking for partners in the searching area according to the glowing characteristics of fireflies, and then moving towards the brighter firefly. Regarding points in the solution set as fireflies, the searching process in solution space is viewed as attraction and movements of fireflies. After many times of movements, all individuals will be gathered in the position with the highest brightness of fireflies, so as to achieve optimization. The process of optimization of the firefly intelligent algorithm is simple and efficient, and therefore is widely applied to functional optimization and combinatorial optimization.

Combining the advantages of the SHC algorithm and the optimization ability of the FA algorithm noted above, it is naturally to adopt the FA to improve the SHC algorithm. Using the FA algorithm to find the optimal value of synchronous neighborhood radius will be more efficient and accurate than the basic hierarchical search do. In addition it is applicable to arbitrary data distribution.

The paper is organized as follows: in Section "Materials and method", basic concepts and principles are introduced firstly; secondly the proposed model of clustering is discussed, and then the flow chart is listed, along with the time complexity analysis of the algorithm. Performance and evaluation of the proposed algorithm is shown by comparing with SC and SHC in Section "Results and Discussions". The last Section concludes this research.

## Materials and method

### The SHC algorithm

The phenomenon of synchronization often appears in physics, it can be expressed as follows. Two or more dynamic systems both have their own evolution and mutual coupling. This effect can be either one-way or two-way streets. When meets certain conditions, the output of these systems will eventually converge and completely be equal under the influence of coupling, this process is called synchronization. The Kuramotom model [36,37] is applied widely as the simple model of synchronization behavior, the generalized definition of Kuramotom model is shown as follows:

**Definition 1 **(Generalize Kuramoto model): The Kuramoto model consists of a population of *N *coupled phase oscillators *θ_i_*(*t*) whose dynamics are governed by:

(1) $$\theta_{i}=\omega_{i}+\overset{N}{\underset{j=1}{\sum }}K_{ij}\text{sin}\left(\theta_{j}-\theta_{i}\right)$$

where *ω_i _*is its natural frequencies and is distributed with a given probability density *g*(*ω*).

Each oscillator tries to run independently at its own frequency, while the coupling tends to synchronize it to all the others.

The sync algorithm is a novel approach for clustering inspired by the powerful concept of synchronization. It regards each data object as a phase oscillator, and each dimension coordinates corresponding to a phase value of the oscillator. Each object couples with data objects in its *ε*-neighborhood, where *ε *is the neighborhood radius. In the effect of synchronization coupling, the object's coordinates are transformed constantly, and objects with the same coordinates will be classified eventually to the same cluster, namely synchronization completion. Let *x *∈ *R^d ^*represents an object in the dataset X and *x_i _*be the *i*-th dimension of the object *x*. The transformation formula of coordinate of *x *shows as follows.

(2) $$x_{i}\left(t+1\right)=x_{i}\left(t\right)+\frac{1}{\left|N_{\varepsilon }(x(t))\right|}\underset{y\in N_{\varepsilon }(x(t))}{\sum }\sin(y_{i}\left(t\right)-x_{i}\left(t\right))$$

where ***ε***-neighborhood is defined in **Definition 2 **below.

**Definition 2 **(*ε*-neighborhood): The *ε*-neighborhood radius of an object is a collection of data with distances to the object less than *ε*:

(3) $$N_{\varepsilon }\left(x\right)=\left\{y\in X|dist\left(x,y\right)\leq \varepsilon \right\}$$

where *dist*(*x,y*) is the metric function of distance and the Euclidean distance is often used. If the object *y *∈ *N_ε_*(*x*), *y *is called the *ε*-neighborhood of *x*, denoted by *x *→*_ε _y*. The relationship of *ε*-neighborhood between objects is symmetrical, namely if *x *→*_ε _y *then *y *→*_ε _x*.

For reducing the dynamic interaction time of synchronization of data in the sync algorithm, the concept of neighborhood closures is proposed in SHC algorithm. Objects in a *ε*-neighborhood closure will reach synchronization complete eventually. So it can detect the clusters even if the objects have not yet reached the same coordinates by classifying data in the same neighborhood closures to the same cluster, which reduces the dynamic interaction time of data.

**Figure 1:**
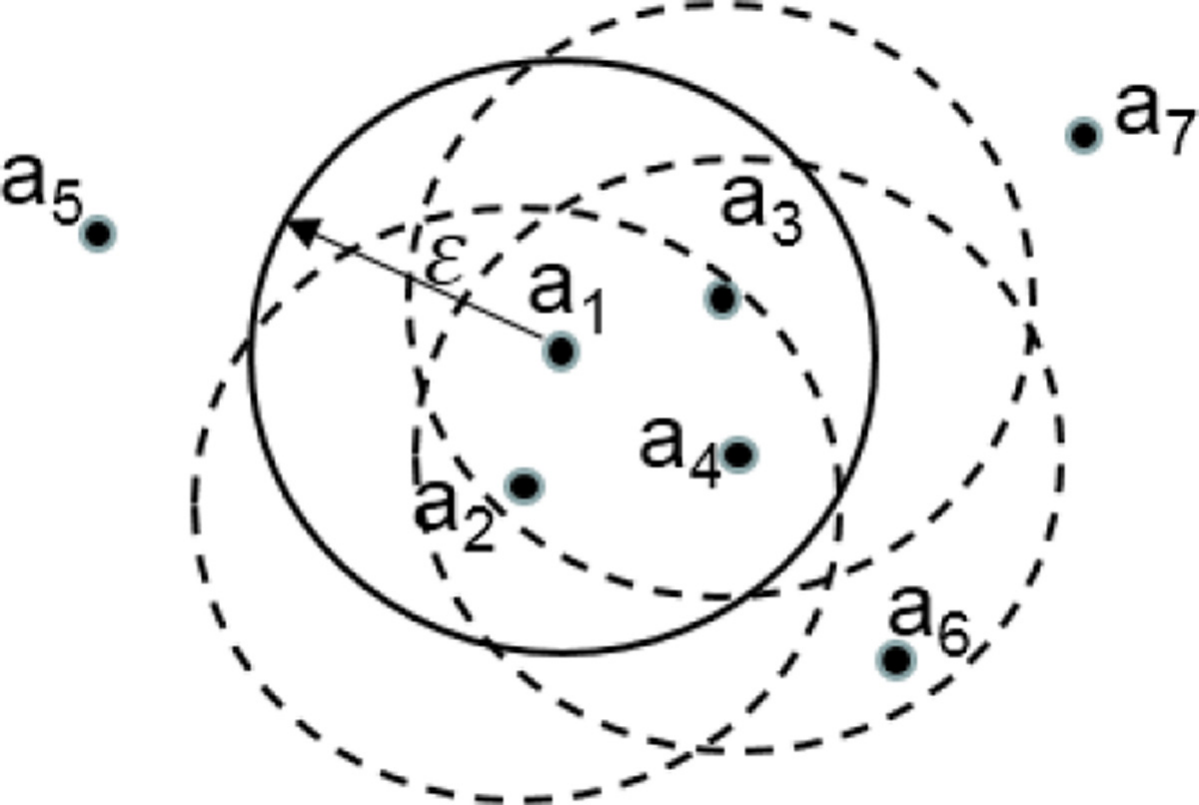
***ε*-neighborhood closures**.

**Definition **3 (*ε*-neighborhood closures): Suppose objects set *X' *⊆ *X*, in the dynamic process of synchronous clustering, if ∀*x*, *y *∈ *X' *satisfies *x *→*_ε _y*, and if ∀*x *∈ *X*, *x *→*_ε _z*, then *z *∈ *X'*, *X'* is called an *ε*-neighborhood closure, that is, for any object *x *∈ *X'*, *N_ε_*(*x*) = *X' *is established.

a_1_, a_2_, a_3_, a_4 _form a *ε*-neighborhood closure in the Figure 1, and will reach complete synchronization eventually.

The optimal value of synchronous neighborhood radius needs to be determined in both the sync algorithm and the SHC algorithm. The SHC algorithm determines synchronous neighborhood radius by means of the hierarchical search that the sync algorithm does. The process of hierarchical search for the optimization of the neighborhood radius shows as follows. Starting in a small neighborhood radius value *ε*, then adding an increment (marked as Δ*ε*) to *ε *at a time (*ε *= *ε *+ Δ*ε*) until the neighborhood radius is large enough to contain all objects. Clustering in each neighborhood radius of *ε*, and it is considered to be optimal when the *ε *gets the best result of clustering.

### The FA

The FA is a random optimization algorithm constructed by simulating the group behavior of the fireflies. There are two important elements in the FA, the light intensity and the attractiveness. The former reflects the advantages and disadvantages of locations of fireflies and the latter determines the movement distances of fireflies attracted. The optimization process of the algorithm is implemented through updating the light intensity and the attractiveness constantly. The mathematical mechanism of the FA is described as follows.

The relative value of the light intensity of fireflies is expressed as:

(4) $$I=I_{0}\times e^{-\gamma r_{ij}}$$

where *I_0 _*is the initial light intensity (*r *= 0) related to the objective function value, the higher the value of objective function is, the stronger the initial light intensity *I_0 _*will be. *γ *is the light absorption coefficient set to reflect the features that the light intensity decreases gradually along with the increase of the distance and the absorption of the medium. It can be set to a constant. *r_ij _*is the space distance between firefly *i *and firefly *j*.

The attractiveness of firefly is expressed as:

(5) $$\beta =\beta_{0}\times e^{-\gamma r_{ij}^{2}}$$

where *β*_0 _is the maximum of attractiveness. *γ *and *r_ij _*are the same as above.

If firefly *i *moves to firefly *j*, the updating of location of firefly *i *is expressed as:

(6) $$x_{i}\left(t+1\right)=x_{i}\left(t\right)+\beta \times \left(x_{j}\left(t\right)-x_{i}\left(t\right)\right)+\alpha \times \left(rand-1/2\right)$$

where *x_i_*(*t*), *x_j_*(*t*) are the space coordinates of firefly *i *and firefly *j *at the time *t*, *α *is step-size in [0, 1], *rand *is a random factor that follows uniform distribution in [0, 1].

Fireflies are distributed to the solution space randomly first of all. Each firefly has its own light intensity according to its location, the light intensity is calculated according to Eq. (4). The firefly with low light intensity is attracted by and moving to the firefly with higher light intensity. The movement distance depends on the attractiveness between them calculated by Eq. (5). The location updating of the fireflies is cumulated based on Eq. (6). There is a disturbing term in the process of updating the location, which enlarges the search area and avoids the algorithm to fall into the local optimum too early. Finally all fireflies will gather in the location of the maximum light intensity.

### The proposed clustering algorithm

The sync algorithm clusters objects based on the principle of dynamic synchronization, which has many advantages in that it reflects the intrinsic structure of the dataset. For example, it can detect clusters of arbitrary number, shape and size and not depend on any assumption of distribution. In addition, it can handle outliers since the noise will not synchronize to cluster objects. However, the running time of the algorithm consists of two parts primarily: The dynamic interaction time of synchronization of data and the process of determining the optimal value of synchronous neighborhood radius, which is too long to process large-scale data.

Aiming to reduce the dynamic interaction time of the sync algorithm, the concept of *ε*-neighborhood closures is proposed in the SHC algorithm. It classifies objects in the same neighborhood closures to a cluster even if objects have not yet reached the same coordinate, which enhances the efficiency of the algorithm by reducing the time of dynamic interaction of data. However, the SHC algorithm determines synchronous neighborhood radius by means of hierarchical search that the sync algorithm does. The hierarchical search for synchronous neighborhood radius not only has low efficiency but also has two shortcomings. Firstly, the hierarchical search is very difficult to find the optimal value of synchronous neighborhood radius in a fixed increment. Secondly, the increment Δ*ε *needs to be adjusted according to different data distributions. For example, in the SHC algorithm, the initial value of *ε *is set to the average distance of all objects of its three nearest neighbors. The increment Δ*ε *is the different value of the average distance of all objects to its four nearest neighbors minus the average distance of all objects to its three nearest neighbors. So the running time of the SHC algorithm is very huge when the dataset is uniform and dispersive. In addition, we must set Δ*ε *small when the data distribution is approximate, otherwise it is hard to find the optimal value of synchronous neighborhood radius.

The FA is a swarm intelligent optimization algorithm developed by simulating the glowing characteristics of fireflies, which is speedy and precise in the optimization process. Using the firefly algorithm to search for the optimal neighborhood radius of synchronous can overcome the drawbacks of the hierarchical search. It adopts fewer searching steps for the optimal value of synchronous neighborhood radius and gets more accurate results than the hierarchical search due to its intelligent searching strategies. So it saves time on determining the optimal value of synchronous neighborhood radius. In addition, it is applicable to any data distributions. So we improve the SHC algorithm by means of the FA and apply the proposed algorithm to clustering PPI data.

#### Preprocessing of PPI data

The PPI data is expressed as a graph, called PPI network, in which each node represents a protein and the edge between two nodes represent the interaction between proteins. In that way, we get an n*n adjacency matrix of nodes. However, the dimension of the adjacency matrix is too big to deal with. Inspired by the spectral clustering, we use the following way to reduce the dimension of the adjacency matrix of PPI.

First, a similarity matrix ***A ***of nodes is constructed as follow.

(7) $$A_{ij}=\left\{\begin{array}{cc} \eta \frac{|N_{i}\cap N_{j}|+1}{min\left(N_{i},N_{j}\right)}+\left(1-\eta \right)\frac{\sum_{k\in I_{ij}}w\left(i,k\right)\cdot \sum_{k\in I_{ij}}w\left(j,k\right)}{\sum_{s\in N_{i}}w\left(i,s\right)\cdot \sum_{t\in I_{j}}w\left(j,t\right)} & i\neq j\\ 0, & i=j \end{array}\right)$$

where *N_i_*, *N_j _*are neighbor nodes of nodes *u *and *v *respectively. *I_ij _*is the common neighbors of *i *and *j*, *w*(*i,j*) is the weight between *i *and *j *to measure the interaction strength, and *η *is constant between 0 and 1.

Eq. (7) considers two aspects of the aggregation coefficient of edges and the weighted aggregation coefficient of edges [38-40]. The first half of Eq. (7) is the aggregation coefficient of edges based on degree, which is portrayed by means of the ratio between adding 1 to the number of common neighbors of two protein nodes and minimal value of the number of neighbors of two nodes. The second half of Eq. (7) is the weighted aggregation coefficient of edges, which is illustrated by the ratio between the product of summation of weight values of edges respectively connecting these two nodes (*i, j*) with their common neighbors (*k*) and the product of summation of weight values of edges linking these two nodes (*i, j*) with their corresponding neighbors (*s, t*). In addition, we use *η *to balance the weight of the two parts.

Then constructing Laplacian matrix ***L ***of matrix ***A***, the ***D ***is the diagonal matrix in which (*i, i*)-element is the sum of ***A***'s *i*-th row.

(8) $$L_{ij}=\left\{\begin{array}{cc} 0 & D_{ii}=0|D_{jj}=0\\ \frac{A_{ij}}{\sqrt{D_{ii}D_{jj}}} & else \end{array}\right)$$

Matrix ***X ***consists of eigenvectors of matrix ***L***'s corresponding to the first three eigenvalues and *X *is normalized. ***X ***is an n*3 matrix, in which lines represent the protein objects (corresponding to the protein nodes in PPI network) and columns are the three-dimensional space coordinates of the protein objects. Our proposed clustering algorithm is calculated based on ***X***.

#### Design of solution space

The solution space of the position of the firefly corresponds to the neighborhood radius of synchronization. The initial light intensity *I*_0 _of one firefly is assigned by the calculation result of objective function, see Eq.(9), which is expressed as the evaluation of clustering results based on the neighborhood radius of the firefly. Moving to the firefly with higher light intensity is regarded as to search for the optimal value of synchronous neighborhood radius. The position of the firefly with the highest light intensity means the optimal value of synchronous neighborhood radius.

#### Definition of objective function

We choose the following object function to evaluate the clustering results. Clusters with higher value of the objective function mean the stronger modularity of clusters, namely, a better clustering result.

(9) $$fval=\sum_{i=1}^{x}\left\{{\left(2\cdot m_{H_{i}}/\left(n_{H_{i}}+m_{H_{i}}\right)\right)}^{\rho }\cdot {\left(\underset{u,v\in H_{i},w\left(u,v\right)\in W}{\sum }w\left(u,v\right)/\underset{u,v\in H_{i},w\left(v,k\right)\in W}{\sum }w\left(v,k\right)\right)}^{1-\rho }\right\}$$

Where *m_H _*is the number of edges that connect points in the cluster *H_i _*, *n_H _*is the number of edges that connect points in the cluster *H_i _*with points out of the cluster *H_i_*, *w*(*u,v*) is the weight between point *u *and point *v*, *x *is the number of clusters, *W *is the set of connections.

The first half of the Eq. (9) is the summation of the ratio of its in-degree to the sum of its in-degree and its out-degree, the second part is the summation of the ratio of its weighted in-degree to the sum of its weighted in-degree and its weighted out-degree. The two parts calculate modularity respectively. We can change the proportion of two parts by adjusting the parameter *ρ*.

#### Flow chart of the algorithm

**Figure 2:**
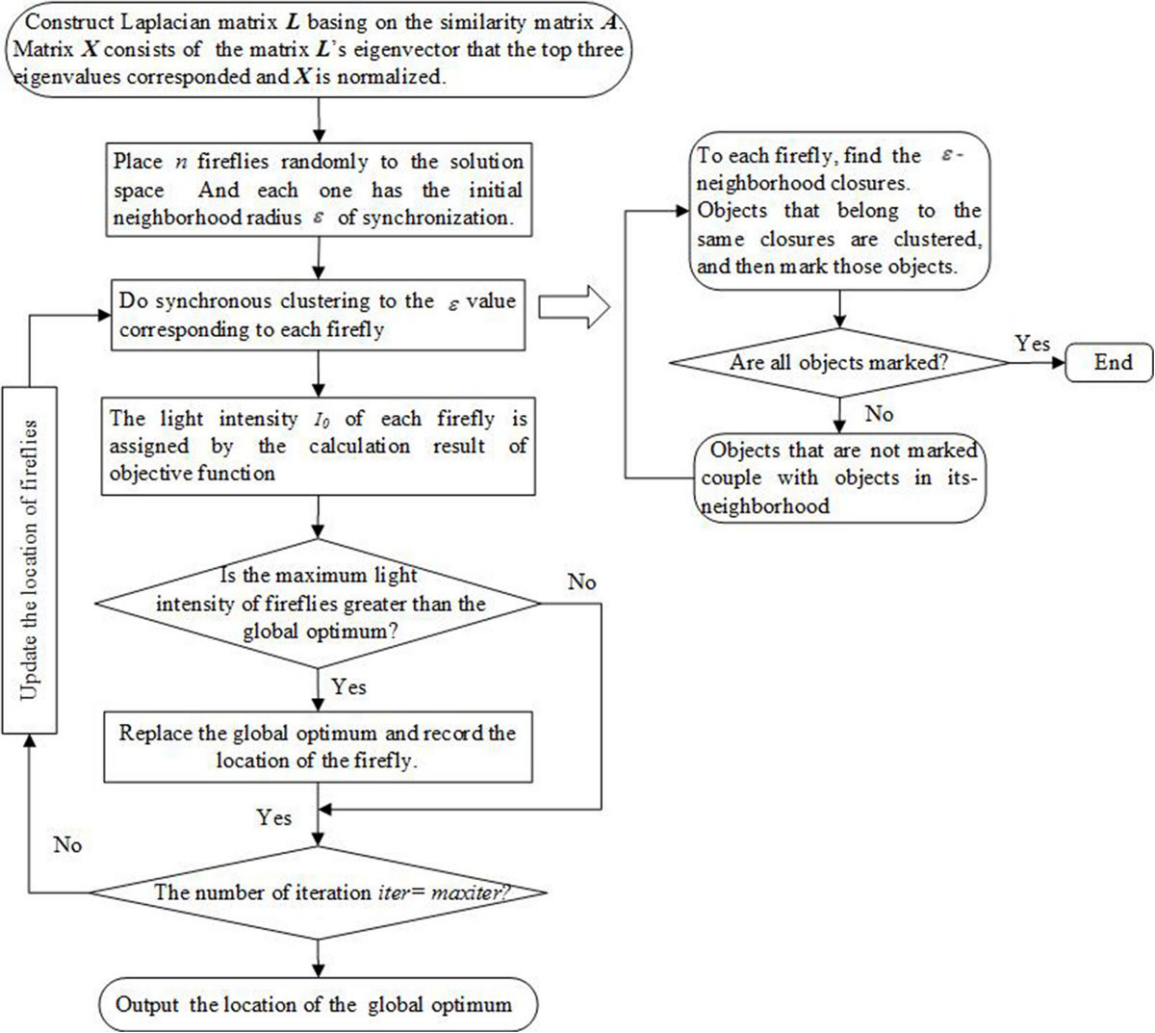
**Flow chart of the improved SHC algorithm**.

Figure 2 is the flow chart of the improved synchronization-based hierarchical clustering algorithm.

The detailed procedures of the improved SHC algorithm are as follows.

**Step 1 **Construct a similarity matrix ***A ***of protein objects, and then get Laplacian matrix ***L ***of the matrix ***A***. Matrix ***X ***consist of the matrix ***L***'s eigenvector that the top three eigenvalues corresponded. ***X ***is an n*3 matrix, in which the rows represent protein objects and the columns are the three-dimensional space coordinates of protein objects.

**Step 2 **The setting of parameters: the number of firefly *N*, the maximum of attractiveness *β*_0_, the light absorption coefficient *γ*, step-size *α*, Maximum iterations *maxiter*, *iter *= 0.

**Step 3 **Initialize the location of firefly in the solution space of the neighborhood radius *ε *of synchronization.

**Step 4 **Do clustering respectively based on the synchronous in *ε *that each firefly corresponding.

**Step 4.1 **Find *ε*-neighborhood closures of protein objects of matrix ***X***. Objects that belong to the same closures are divided into a cluster, and then mark those objects.

**Step 4.2 **If all points are marked, return to the result of clustering, otherwise the unmarked objects couple with the objects in its *ε*-neighborhood according to the formula (2), and then go to **step 4.1**.

**Step 5 **The light intensity of fireflies are assigned by the calculation result of the objective function (9) according to the clustering result. Compare the brightness of fireflies, if *I_i _*>*I_i_*, calculate the attractiveness according formula (5), and then update the location of firefly *i *according to the formula (6).

**Step 6 ***iter *= *iter*+1;

**Step 7 **If *iter *<= maxiter, *go *to **Step 4**, otherwise output the clustering result that the firefly with the highest light intensity.

#### The time complexity of algorithm

The time complexity of the SHC algorithm is *O*(*T*·*n*^2) in a certain neighborhood radius, where *n *is the number of nodes, *T *is the number of synchronization to form *ε*-neighborhood closures. Assuming that the number of dynamic interaction of synchronization of data to form *ε*-neighborhood closures will not change in different neighborhood radiuses, the time complexity of the SHC algorithm is *O*(*k*·*T*·*n*^2), *k *is the number of iterations of searching for the optimal *ε*-neighborhood radius based on the hierarchical search. Replacing the hierarchical search with FA results in the decrease of *k*, it enhances the efficiency of the algorithm.

## Results and discussions

### Analysis of experiment parameters

We set the weigh *η *= 0.5 to balance the aggregation coefficient of the edge and the weighted aggregation coefficient of the edge in Eq. (8) in the preprocessing. The setting of parameters of the FA: the maximum of attractiveness *β*_0 _= 1, the light absorption coefficient *γ *= 1, the step-size *α *= 0.9, we set *ρ *= 0.8 in the objective function.

The value of *ρ *in the objective function of the FA is important to evaluate the result of clustering. Aiming at reflecting the correlation between *fval *and *f-measure *in different *ρ*, we calculate the Pearson correlation coefficient of *fval *and *f-measure *in 20 values that distribute evenly in the region of 1 to 6 of the neighborhood radiuses. The result is shown in Table 1 the *fval *and *f-measure *correlation is extremely linear when *ρ *= 0.8. Thus we set *ρ *= 0.8.

**Table 1:** Comparisons of the Pearson correlation coefficient between *fval *and *f-measure *in different *ρ *

*ρ*	0	0.1	0.2	0.3	0.4	0.5	0.6	0.7	0.8	0.9	1
*r*	50.9059	51.9654	52.0163	52.0588	52.0933	52.1201	52.1396	52.1521	52.1580	52.1574	52.1508

The Pearson correlation coefficient is shown as:

(10) $$r=\frac{1}{n-1}\overset{n}{\underset{i=1}{\sum }}\left(\frac{X_{i}-\bar{X}}{S_{X}}\right)\left(\frac{Y_{i}-Ȳ}{S_{Y}}\right)$$

Where $\bar{X}$, $\bar{Y}$ and *S_X_*, *S_Y _*represent the mean value and the variance of *X *and *Y*, respectively.

With practical consideration, we set the searching number for the optimal threshold of the neighborhood radius of synchronization small values. As the value of step-size *α *in FA is important to the result, we calculate 20 times to get the average value of the maximum objective function in different *α*, which is shown in Table 2. Tests are carried on *n *= 6, *maxiter *= 30 cases. The average of the maximum objective function value is optimal when *α *= 0.9. Thus we set step-size α = 0.9.

**Table 2:** The average of maximum objective function values of different *α *for 20 times clustering (*value*)

*α*	0	0.1	0.2	0.3	0.4	0.5	0.6	0.7	0.8	0.9	1
*value*	94.4592	95.7940	95.4177	95.4546	95.7102	96.1161	95.2840	95.7408	95.7408	96.2709	95.7664

### The performance comparison on different optimization algorithms

In the experiments, the dataset of PPI networks was downloaded from MIPS database [40], which consists of two sets of data: one is the experimental data which contains 1376 protein nodes and the 6880 interactive protein-pairs, which is considered the training dataset; the other describes the result that the proteins belong to identical functional module, which is regarded as the standard dataset [41], containing 89 clusters.

**Figure 3:**
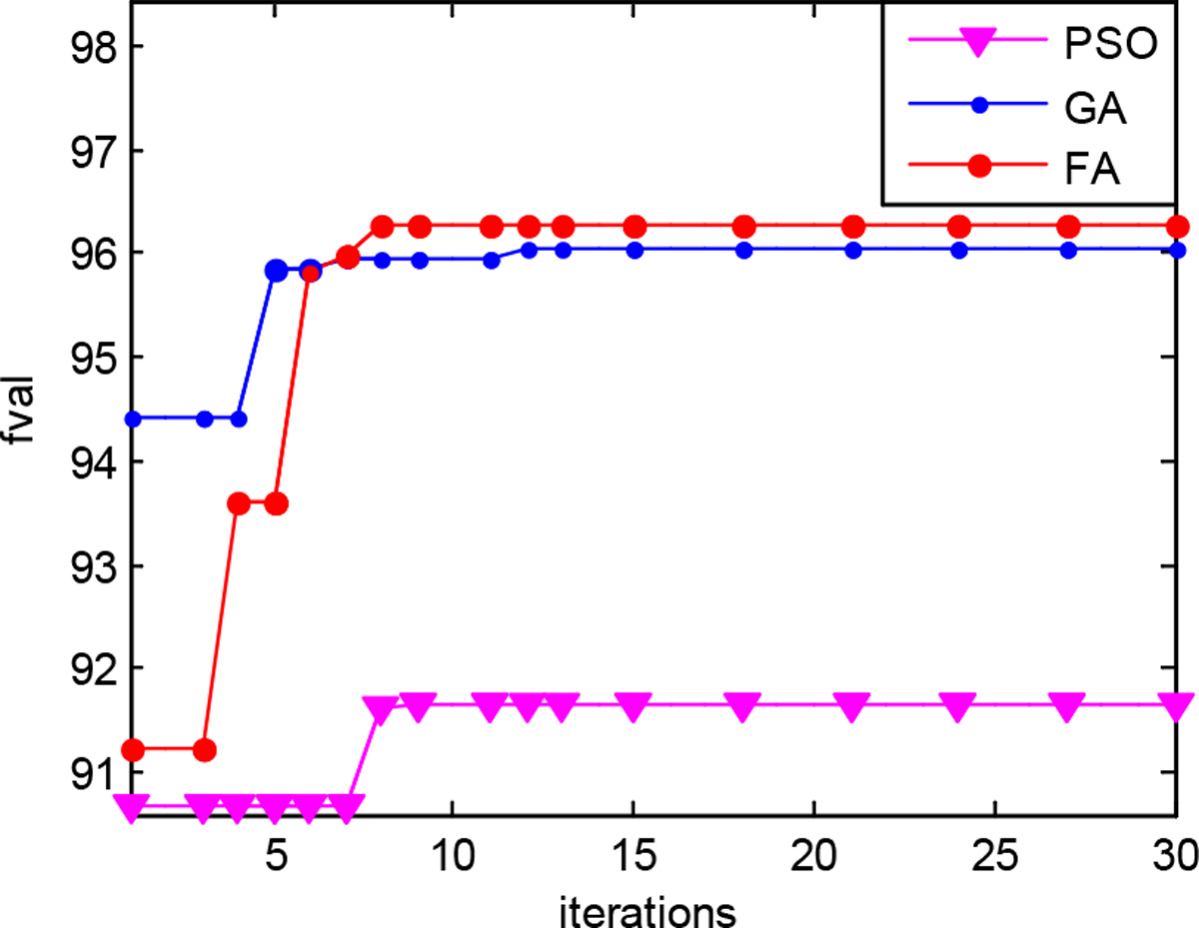
**Plots of the optimal objective function value with the number of iterations of the FA, PSO and GA**. (a) Comparison of *precision *value (b) Comparison of *recall *value (c) Comparison of *f-measure *value

Inspired by the swarm optimization algorithms [42-45], we use them to search for the optimal threshold of the neighborhood radius of synchronization. The experimental parameters of PSO, GA and FA algorithms are shown in Table 3. The parameters of PSO and GA are set empirically based on the references [46,47]. We also calculate the maximum objective function values for 10 times and the average of the maximum objective function value over 20 times of the FA, the PSO, and the GA, which is shown in Table 4 and Table 5. The plots of the optimal objective function value with the number of iterations are depicted in Figure 3. The FA algorithm always converges to the optimal value fast. However, the PSO algorithm falls into a low value sometimes, the GA algorithm gets a higher value always. The FA performs best when considering convergence speed and global optimization ability comprehensive.

**Table 3:** The experimental parameters of PSO, GA and FA algorithms

PSO	The global acceleration coefficient (*c_1_*) = 2	The local acceleration coefficient (*c_2_*) = 2
GA	The crossover probability (*pcro*) = 0.8	The mutation probability (*pmut*) = 0.085

FA	The maximum of attractiveness *β*_0 _= 1 The step-size *α *= 0.9	The light absorption coefficient *γ *= 1

**Table 4:** The maximum objective function value of 10 times (*value*) of the FA, PSO, and GA algorithms

Algorithm	1	2	3	4	5	6	7	8	9	10
PSO	96.2709	96.2709	91.6680	96.2709	96.2709	91.6680	91.6680	96.2709	96.2709	96.2709

GA	96.2709	95.8277	95.9618	96.1602	96.0500	95.9618	95.9655	96.0500	96.2709	96.2709

FA	96.2709	96.2709	96.2709	96.2709	96.2709	96.2709	96.2709	96.2709	96.2709	96.2709

**Table 5:** The average of the maximal objective function value of PSO, GA and FA algorithms on 10 times

Algorithm	PSO	GA	FA
*Value*	94.8900	96.0790	96.2709

*Precision, recall*, and *f-measure *are employed as the metric for clustering in this study. *Precision *[48] is the ratio of the number of maximum matching nodes in training with standard database to the number of training nodes. *Recall *[48] is the ratio of the largest number of nodes in training matched the standard database to the number of nodes in the standard database. *Precision *and *recall *are defined as Eqs. (11)- (12), respectively while *F-measure*, the harmonic mean of *precision and recall *is defined as Eq. (13).

(11) $$precison\left(C\text{|}F\right)=\frac{MMS\left(C,F\right)}{\left|C\right|}$$

(12) $$recall\left(C\text{|}F\right)=\frac{MMS\left(C,F\right)}{\left|F\right|}$$

(13) $$f\text{ - }measure=\frac{precision\cdot recall}{precision+recall}$$

where *C *is the set of cluster results of training database, *F *stands for the set of cluster results of MIPS database, |*C*| represents the number of cluster nodes in training database, |*F*| represents the number of cluster nodes in standard database, *MMS *represents the number of maximum matching nodes in training database with standard database.

The running time of SHC algorithm and the proposed algorithm are proportional to the searching number of times, when the searching number of times is big, the algorithms are impractical. So we compare the two algorithms in 20 times searching. The proposed method is compared with SC algorithm and the SHC algorithm in terms of *precision*, *recall *and *f-measure*, respectively.

**Figure 4:**
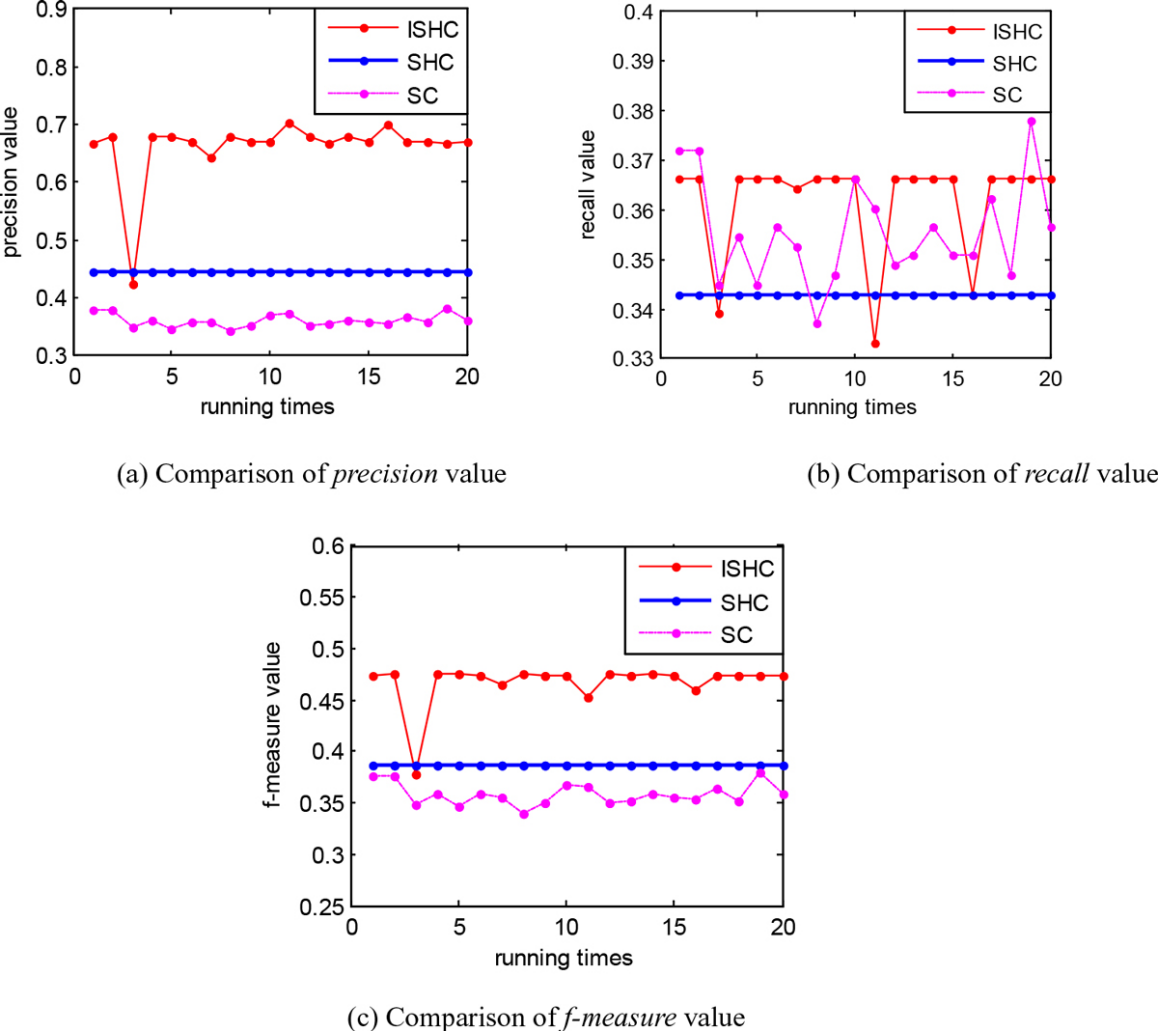
**The improved algorithm compared with spectral clustering algorithm and SHC in *precision*, *recall *and *f-measure***.

The ISHC algorithm converges to the optimal threshold of the neighborhood radius of synchronization steadily when the searching number is large in Table 4. In order to obtain a more intuitive and obvious result, we reduce the searching number in Figure 4. We can see from the Figure 4 andTable 6 that the performance of the ISHC algorithm is better than the SC algorithm and the SHC algorithm in *precision*, *recall *and *f-measure*. We compare our proposed algorithm with the SHC algorithm in 20 times searching as in Figure 4, the result of our proposed algorithm is not stable sometimes. We also compare our proposed algorithm with some classical algorithms shown in Table 6. In these algorithms we listed, the result of our proposed algorithm performs best.

**Table 6:** Comparison of *precision, recall *and *f-measure *among ISHC, SHC, SC and other algorithms

Algorithm	*precision*	*recall*	*f-measure*
SHC[31,32]	0.4447	0.3430	0.3873
SC[13]	0.3612	0.3555	0.3584
MCL[12]	0.3569	0.3879	0.3717
Newman[8]	0.4665	0.4186	0.4413
RNSC[9]	0.4067	0.4696	0.4359
ISHC	0.6624	0.3620	0.4673

## Conclusion

The sync algorithm is a novel clustering algorithm based on the model of synchronous dynamics, which can detect clusters with arbitrary shape and size and has the anti-noise ability. However, the running time of the algorithm consists of two parts primarily: The dynamic interaction time of synchronizing data and the process of determining the optimal synchronous neighborhood radius, which is too long to process large-scale data. The SHC algorithm proposes the concept of neighborhood closures reducing dynamic interaction time of the sync algorithm. In our proposed algorithm, the efficiency and accuracy is further improved by using the FA to determine the optimal thresholds of neighborhood radius of synchronization. The *recall*, *precision *and *f-measure *of our proposed algorithm are improved compared with SC and SHC algorithms. In future, we are intending to seek a more suitable model of synchronous dynamics for PPI data clustering to improve the effect of the algorithm, also the time complexity is still need to be decreased.

## Competing interests

The authors declare that they have no competing interests.

## Declarations

The funding for the publication charges comes from: the National Natural Science Foundation of China (61100164, 61173190), Scientific Research Start-up Foundation for Returned Scholars, Ministry of Education of China ([2012]1707) and the Fundamental Research Funds for the Central Universities, Shaanxi Normal University (GK201402035, GK201302025).

This article has been published as part of *BMC Genomics *Volume 16 Supplement 3, 2015: Selected articles from the 10th International Symposium on Bioinformatics Research and Applications (ISBRA-14): Genomics. The full contents of the supplement are available online at http://www.biomedcentral.com/bmcgenomics/supplements/16/S3.
